# AKT Signaling Regulates Agrin-Mediated Acetylcholine Receptor Surface Density

**DOI:** 10.3390/medicina62030456

**Published:** 2026-02-27

**Authors:** Natasha Jaiswal, Nathan Roger Lin, Colton Frank Lehnen, Amaan Tariq, Laura Lynn Lukov, Chi Zhang

**Affiliations:** 1 Department of Health and Kinesiology, Purdue University, West Lafayette, IN 47907, USA; 2 Department of Biomedical Health Sciences, Purdue University, West Lafayette, IN 47907, USA; 3 Department of Chemistry, Purdue University, West Lafayette, IN 47907, USA

**Keywords:** acetylcholine receptor, AKT signaling, Agrin, proteasome pathway, skeletal muscle

## Abstract

*Background and Objectives:* Acetylcholine receptors (AChRs) are ligand-gated ion channels concentrated at the postsynaptic membrane of skeletal muscle fibers, where their abundance is essential for efficient neuromuscular transmission. The serine/threonine kinase AKT is a central signaling node in muscle homeostasis, regulating metabolism, growth, and survival. However, its role in the Agrin-mediated regulation of postsynaptic AChRs remains incompletely defined. Here, we demonstrate a novel role of AKT in regulating Agrin-induced AChR accumulation in differentiated C2C12 myotubes. *Materials and Methods:* Differentiated C2C12 myotubes were stimulated with Agrin in the presence or absence of the AKT inhibitor MK2206 during either the formation or maintenance phase. AChR clustering was quantified using α-bungarotoxin labeling. Expression of AChR subunits and neuromuscular junction-associated genes was assessed. Proteasome involvement was examined using the inhibitor MG132. *Results:* Pharmacological inhibition of AKT using MK2206 during either the formation or maintenance phase of Agrin stimulation significantly reduced α-bungarotoxin-labeled AChR intensity. AKT inhibition also attenuated Agrin-induced expression of multiple AChR subunits and neuromuscular junction-associated genes. Importantly, inhibition of proteasome activity with MG132 restored AChR intensity in the presence of AKT inhibition, suggesting that AKT signaling limits proteasome-dependent AChR loss. *Conclusions:* these findings identify AKT as a regulator of Agrin-mediated AChR accumulation and maintenance in vitro. These findings identify AKT as a critical integrator of metabolic and synaptic signaling required for postsynaptic receptor stability, with implications for neuromuscular disorders and muscle atrophy.

## 1. Introduction

The neuromuscular junction (NMJ) is a specialized synapse where motor neuron terminals communicate with skeletal muscle fibers to initiate contraction. As one of the earliest synaptic structures established during mammalian development, the NMJ is indispensable for voluntary movement, posture control, and respiratory function [1]. Proper formation and maintenance of this interface are critical for neuromuscular performance, and its deterioration contributes to age-related sarcopenia, denervation-induced atrophy, and inherited disorders, such as Duchenne muscular dystrophy (DMD) [2,3]. Although the developmental mechanisms governing NMJ assembly are well characterized, the molecular pathways that preserve mature NMJ structure and function throughout life remain less clearly defined.

The clustering of nicotinic acetylcholine receptors (AChRs) at the postsynaptic membrane is a hallmark of the NMJ [4]. This initial step in receptor clustering orchestrates the postsynaptic differentiation through a network of scaffolding and adaptor proteins [5,6]. Interestingly, while motor neurons have traditionally been viewed as the principal drivers of NMJ formation, an expanding body of evidence demonstrates that muscle-derived factors also play essential roles in synaptic organization [7,8,9]. For instance, aneural “pre-patterns” of AChR clusters that arise on nascent myotubes can serve as guidance cues for approaching motor axons [10]. Likewise, the muscle-specific receptor tyrosine kinase MuSK and its co-receptor, low-density lipoprotein receptor-related protein 4 (LRP4), not only mediate Agrin-induced AChR clustering but also transmit retrograde signals that influence presynaptic differentiation [11,12]. Importantly, studies in *Drosophila* suggest that some components involved in early NMJ assembly may later contribute to synaptic remodeling and AChR turnover [13,14]. However, the intracellular mechanisms that maintain receptor stability within mature muscle remain largely unknown. In particular, the contribution of muscle-intrinsic signaling pathways to the long-term stability of AChR clusters is poorly understood.

AKT, also known as protein kinase B, is a key downstream effector of phosphoinositide 3-kinase (PI3K) that integrates multiple extracellular cues such as insulin, insulin-like growth factor 1 (IGF-1), and mechanical stimulation, including exercise. In skeletal muscle, AKT controls growth and metabolic adaptation by coordinating the activating mechanistic (or mammalian) Target of Rapamycin (mTOR) signaling and repressing *Forkhead box O* (a subfamily of transcription factors) (FOXO)-mediated atrophy pathways [15,16,17]. Beyond its well established role in muscle growth, our recent work demonstrates that AKT activity promotes mitochondrial biogenesis, oxidative metabolism, and fiber-type transitions that underlie endurance adaptations. Conversely, loss of AKT signaling results in glycolytic remodeling, reduced exercise tolerance, and heightened susceptibility to muscle wasting. AKT is also indispensable for disuse-induced muscle atrophy [18]. Interestingly, AKT levels are strongly regulated by denervation or injury [8,19]. Consistent with the ability to promote a trained phenotype even in inactive muscle, a defect in AKT activity is associated with different contexts of muscular wasting, including Duchenne muscular dystrophy [20,21], denervation-induced fiber atrophy, statin-mediated muscle dysmorphology, sarcopenia [22] or amyotrophic lateral sclerosis [23]. Despite the central role of AKT in skeletal muscle homeostasis and its strong correlation with neuromuscular disease conditions, the role of skeletal muscle AKT in NMJ organization, particularly in AChR clustering and stability, remains unexplored.

Several lines of evidence indirectly implicate AKT as a potential regulator of NMJ stability. Denervation and neuromuscular diseases are characterized by altered AKT activity, transcriptional remodeling of NMJ-associated genes and accelerated AChR turnover [19,22,23]. Furthermore, AKT–mTOR signaling has been shown to localize to the postsynaptic region and regulate synapse-associated gene expression during aging and denervation [3,19]. Despite these observations, it remains unknown whether muscle-intrinsic AKT signaling directly regulates Agrin-mediated AChR cluster formation or the maintenance of postsynaptic receptor stability. Addressing this gap is critical for understanding how metabolic and growth factor signaling pathways intersect with synaptic maintenance mechanisms in skeletal muscle.

Here, we define a novel role for AKT in regulating both the formation and maintenance of Agrin-induced AChR clusters in vitro. Using pharmacological AKT inhibitor MK2206 in C2C12 myotubes, we demonstrate that AKT is required for transcriptional activation of AChR subunits and NMJ-associated genes, as well as for protecting AChR clusters from proteasome-dependent degradation. Together, these findings identify AKT as a central node linking growth factor signaling with synaptic receptor stability, providing new mechanistic insight into how muscle-intrinsic mechanisms sustain the postsynaptic compartment.

## 2. Materials and Methods

### 2.1. Cell Culture and Differentiation

Murine C2C12 myoblast cell lines (American Type Culture Collection (ATCC); Manassas, VA, USA) were maintained under standard culture conditions in high-glucose Dulbecco’s Modified Eagle Medium (DMEM) supplemented with 20% fetal bovine serum (FBS). Cells were grown at 37 °C in a humidified atmosphere containing 5% carbon dioxide until the cells reached 90% confluence. For differentiation, the growth medium was replaced with the differentiation medium (DMEM containing 2% horse serum) for 6–7 days. Media was refreshed every 24 h to promote the formation of multinucleated myotubes. Fully differentiated myotubes were used for the subsequent experimental treatment.

### 2.2. AChR Cluster Formation Assay

To induce postsynaptic acetylcholine receptor accumulation, differentiated C2C12 myotubes (5–6 days post-differentiation) were pretreated with the AKT inhibitor (MK2206 dihydrochloride, MCE MedChemExpress, Monmouth Junction, NJ, USA, Cat. No.: HY-10358, dissolved in 100% DMSO). Following 5–6 days of differentiation, cells were treated with 10 μM MK2206 for 30 min prior to the addition of 1 nM human recombinant Agrin (MCE MedChemExpress, Monmouth Junction, NJ, USA, Catalog No. HY-P79236) for 16 h. Cells were maintained at 5% CO2 and 37 °C. Following 16 h incubation, cells were fixed in 2% paraformaldehyde in PBS for 15 min at 37 °C. After 15 min, the fixing solution was removed, and the cells were washed with PBS three times for 5 min and blocked with 5% BSA. AChR cluster intensities were visualized with tetramethylrhodamine-conjugated a-bungarotoxin (Invitrogen, Carlsbad, CA, USA, Catalog No. T1175). Coverslips were mounted using VECTASHIELD Antifade Mounting Medium with DAPI to counterstain nuclei (Thermo Scientific Fisher, Waltham, MA, USA, Catalog No. NC9524612). Images were acquired using an LSM 510 confocal microscope at 40× magnification. The 405 nm laser was used to excite fluorescence signals from DAPI, which were acquired at 420 nm long pass filters and a photomultiplier tube. For TRITC–α-bungarotoxin, a 543 nm laser was used for excitation, and a 560 nm long pass filter plus a photomultiplier was used for acquisition. A minimum of 10 microscopic images per experimental condition were acquired. All images were acquired using identical acquisition settings and a 40× objective to ensure consistent field size and unbiased quantitative comparison across experimental conditions. Because the primary endpoint of this study was AChR surface abundance, quantified by α-bungarotoxin fluorescence intensity, imaging parameters were optimized for reliable intensity measurements rather than ultrastructural resolution of NMJ morphology. Images were analyzed manually using ImageJ software (Version 1.54p).

### 2.3. AChR Cluster Stability Assay

C2C12 myotubes (5–6 days post differentiation) were incubated with 1 nM human recombinant Agrin (MCE MedChemExpress, Monmouth Junction, NJ, USA, Cat. No.: HY-P79236) for 16 h. AKT activity is inhibited using 10 μM MK2206 during the final 3 h or 6 h of 16 h Agrin treatment. For proteasome inhibition, myotubes were treated with MG132 (10 µM; Catalog No. BML-PI102-0005) 30 min prior to MK2206 administration. Following MK2206 treatment for 3 h or 6 h, cells were washed, fixed in 2% paraformaldehyde, blocked for 1 h in 5% BSA and stained with tetramethylrhodamine-conjugated α-bungarotoxin for 1 h and counterstained for nuclei with DAPI containing Vecta-shield. All images were acquired using identical acquisition settings and a 40× objective to ensure consistent field size and unbiased quantitative comparison across experimental conditions. Qualitative analysis focused on changes in surface AChR fluorescence intensity as a measure of receptor stability, rather than detailed ultrastructural features of AChR clusters.

### 2.4. RNA Extraction and Quantitative PCR (qPCR)

Total RNA was extracted from myotubes using TRIzol reagent (Invitrogen, Waltham, MA, USA), as described previously [18,24]. cDNA was synthesized using the RevertAid First Strand cDNA synthesis Kit (Thermo Fisher, Waltham, MA, USA, Catalog No. K1621). Quantitative PCR was performed using LightCycler 480 SYBR Green I Master (Thermo Fisher, Waltham, MA, USA, Catalog No. A46111) on a Roche Real-Time PCR system. Relative expression levels were normalized to TBP-1 using the ΔΔCp method. Primers were designed for AChR subunits (α, δ, and ε), NMJ-associated genes (MuSK, LRP4, Agrin, and Utrophin), and proteasome-related genes (*Psma1*, *Psmc4*, and *Psmd11*).

### 2.5. Protein Extraction and Immunoblotting

Cells were lysed in RIPA buffer containing protease and phosphatase inhibitors (Thermo Fisher). Protein concentrations were determined by BCA assay (Pierce, Appleton, WI, USA). Equal amounts of protein were separated on SDS-PAGE gels and transferred to PVDF membranes (Millipore, Burlington, MA, USA). Membranes were blocked with intercept (TBS) Blocking Buffer (LICOR, Lincoln, NE, USA) in TBS-T and incubated overnight at 4 °C with primary antibodies from Cell Signaling Technology against phospho-AKT (Ser473) (#4060L), phospho-S6 (Ser240/244) (#5364), phospho-PRAS40 (Thr246) (#C77D7), and HSP90 (#4874S). After washing, membranes were incubated with IRDye 800CW Donkey anti-Goat IgG Secondary Antibody (1:10,000; LICORbio, Lincoln, NE, USA) and visualized using LICOR.

### 2.6. Statistical Analysis

All statistical analyses were performed using GraphPad Prism 10 version 10.6.1. Data are presented as mean ± Standard Error of the Mean (SEM). Two-way analysis of variance (ANOVA) followed by Tukey’s test was used. Statistical significance was defined as *p* < 0.05. Fluorescence intensity was quantified using identical analysis parameters across all images to minimize technical variability.

## 3. Results

### 3.1. AKT Is Critical in Agrin-Induced AChR Cluster Intensity in C2C12 Myotubes

**Figure 1:**
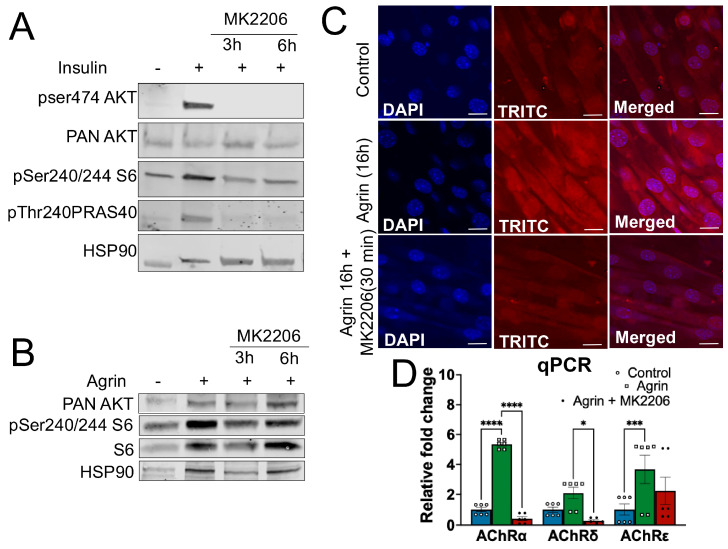
**AKT inhibition suppresses Agrin-induced AChR cluster formation in C2C12 myotubes.** (**A**) Immunoblot analysis showing reduced phosphorylation of AKT (pSer474) and its downstream targets S6 (pSer240/244) and PRAS40 (pThr240 following MK2206 treatment (10 μM) for 3 h or 6 h. (**B**) Western blot analysis of PAN-AKT, pSer240/244-S6, S6 and HSP90 in C2C12 myotubes treated with MK2206 (10 μM, 3 h or 6 h) following Agrin treatment for 16 h. (**C**) Representative images of AChR cluster intensity visualized by fluorescent α-bungarotoxin (α-bungarotoxin) staining in C2C12 myotubes treated with n-Agrin, with or without MK2206 pretreatment. MK2206 markedly reduced the intensity of Agrin-induced AChR clusters. (**D**) mRNA expression of AChR subunits (α, δ, ε) in C2C12 myotubes treated with neural Agrin (n-Agrin, 16 h) with or without MK2206 pretreatment. MK2206 significantly reduced Agrin-induced expression of AChRα and AChRδ and robustly suppressed AChRε expression. Data are presented as mean ± SEM; * *p* < 0.05, *** *p* < 0.001, **** *p* < 0.0001. Images were acquired using a 40× objective; scale bar, 20 µm.

To confirm the efficacy of MK2206 on AKT inhibition, C2C12 myotubes were treated with MK2206 (10 μM, 30 min or 6 h), and AKT signaling was assessed under basal and insulin-stimulated conditions. Insulin stimulation robustly increased AKT phosphorylation at Ser473, whereas MK2206 treatment (for 30 min and 6 h) completely abolished pSer473-AKT in response to insulin-stimulation states (Figure 1A). Consistent with pSer473-AKT inhibition, phosphorylation of the downstream target pSer240/244-S6 and pThr246-PRAS40 was also markedly reduced following MK2206 treatment. These findings confirm potent inhibition of canonical AKT signaling in response to MK2206 without altering myotube morphology.

Agrin, a neural-derived heparan sulfate proteoglycan, is the key extracellular cue driving acetylcholine receptor (AChR) accumulation at the postsynaptic membrane. To induce AChR accumulation in vitro, differentiated C2C12 myotubes were treated with neural Agrin (n-Agrin) for 16 h in the presence or absence of MK2206 pretreatment. Immunoblot analysis revealed no significant changes in pSer473-AKT in response to Agrin stimulation. In addition, slight differences in pSer240/244-S6 band intensity are visually apparent in the Agrin-treated wells; these differences are accompanied by corresponding changes in total S6 and HSP90 (Figure 1B). Densitometric analysis normalized to total S6 and HSP90 did not reveal statistically significant differences among treatments (Supplementary Figure S1A), indicating that Agrin alone does not measurably activate canonical AKT signaling in C2C12 myotubes.

To assess the functional consequences of AKT inhibition on Agrin-induced AChR accumulation, surface AChR intensity was visualized using fluorescent α-bungarotoxin (TRITC–α-bungarotoxin) in non-permeabilized C2C12 myotubes. Consistent with the previous studies, Agrin treatment markedly increased AChR-associated fluorescence intensity compared to unstimulated controls. However, pretreatment of C2C12 myotubes with the AKT inhibitor MK2206 for 30 min significantly attenuated this response (Figure 1C and Supplementary Figure S1B), demonstrating that AKT activity is required for optimal Agrin-mediated receptor accumulation.

Because AChR accumulation at the neuromuscular junction is partially supported by transcriptional regulation of receptor subunits, we next examined whether AKT inhibition alters Agrin-induced AChR gene expression [8,9,10]. Consistent with the reduced AChR intensity, MK2206 pretreatment significantly attenuated Agrin-mediated induction of *Chrna1* and *Chrnd*, encoding for AChRα and AChRδ, respectively. This was associated with a robust reduction in *Chrne* encoding for the AChRε subunit (Figure 1D). While we examined selected nAChR subunits associated with mature receptor assembly, *Chrnb1* or *Chrng* was not assessed. Given the established roles of the β subunit in receptor trafficking and the γ subunit in developmental NMJ formation, future studies will be required to determine whether these subunits are similarly regulated in this model.

Together, these findings demonstrate that AKT signaling supports Agrin-induced acetylcholine receptor accumulation in C2C12 myotubes, possibly by promoting the transcriptional regulation of AChR subunits, despite Agrin itself not directly activating canonical AKT signaling pathways.

### 3.2. AKT Is Important for the Maintenance of Agrin-Induced AChR Intensity in C2C12 Myotubes

Next, we evaluated the role of AKT in the maintenance of Agrin-induced AChR clusters. Differentiated C2C12 myotubes were treated with neural Agrin for 16 h to induce AChR accumulation. The AKT inhibitor MK2206 was then applied for 3 h or 6 h post-Agrin treatment to specifically assess the requirement for AKT activity during the maintenance phase.

**Figure 2:**
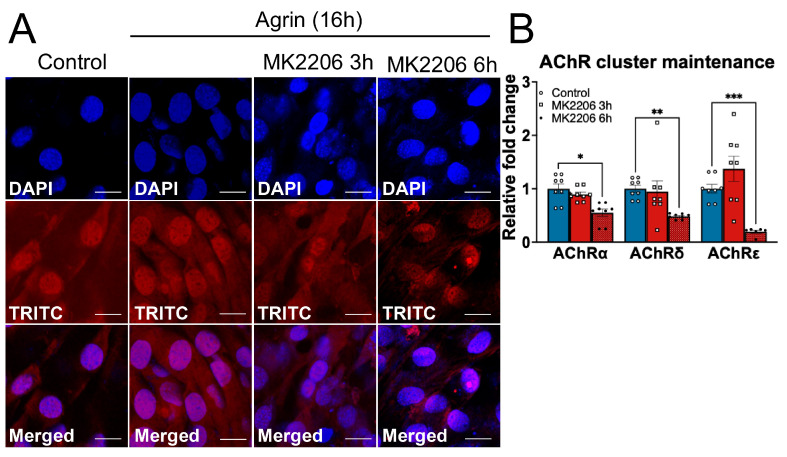
**AKT is required for the maintenance of Agrin-induced AChR cluster intensity.** (**A**) Confocal images of AChR clusters intensity visualized with Alexa547-conjugated α-bungarotoxin in C2C12 myotubes treated with Agrin alone for 16 h or Agrin + MK2206 for 3 h or 6 h. (**B**) qPCR analysis of AChR subunit transcripts (α, δ, and ε) after 3 h or 6 h of MK2206 treatment during the final phase of Agrin stimulation for 16 h. Data are presented as mean ± SEM. * *p* < 0.05, ** *p* < 0.01, *** *p* < 0.001 vs. unstimulated control. Images were acquired using a 40× objective; scale bar, 20 µm.

To determine the functional effect of AKT deficiency on the AchR stability, the expression level of surface AchR protein was assessed using α-bungarotoxin conjugated with Alexa-647 (TRITC-α-bungarotoxin) in a time-dependent manner. Consistent with the previous findings, Agrin treatment alone resulted in a significant increase in AchR protein expression compared to the unstimulated cells, as demonstrated by the enhanced fluorescence intensity (Figure 2A, Panels 1–2 and Supplementary Figure S2A). Interestingly, MK2206 treatment significantly reduced AChR cluster intensity at 3 h, and this reduction became more pronounced after 6 h of treatment (Figure 2A, Panels 3–4 and Supplementary Figure S2A). To determine whether the reduction in AChR fluorescence was associated with transcriptional changes, we measured the mRNA expression of AChR subunits following AKT inhibition during the maintenance phase. Consistent with the protein levels of AChR measured using α-bungarotoxin staining, no significant changes in the transcript levels of *Chrna1*, *Chrnd* and *Chrne* were observed following 3 h of MK2206 treatment in Agrin-stimulated C2C12 cells. However, extending AKT inhibition to 6 h resulted in a significant reduction in these subunits (Figure 2B and Supplementary Figure S2A).

The absence of early transcriptional changes at 3 h, despite reduced surface AChR intensity, suggests that short-term AKT inhibition primarily affects receptor stability or turnover rather than transcription. In contrast, prolonged AKT inhibition appears to impact both receptor maintenance and gene expression.

Together, these findings indicate that sustained AKT activity is required to maintain Agrin-induced acetylcholine receptor accumulation in C2C12 myotubes, supporting a role for AKT in preserving postsynaptic receptor intensity following cluster formation.

### 3.3. AKT Regulates NMJ Gene Transcription During Both the Formation and Maintenance of AChR Clusters In Vitro

To investigate the mechanisms underlying reduced Agrin-mediated AChR accumulation and impaired maintenance following AKT inhibition, we examined the transcriptional regulation of neuromuscular junction (NMJ)-associated genes during both the formation and maintenance phases of Agrin-induced AChR accumulation.

**Figure 3:**
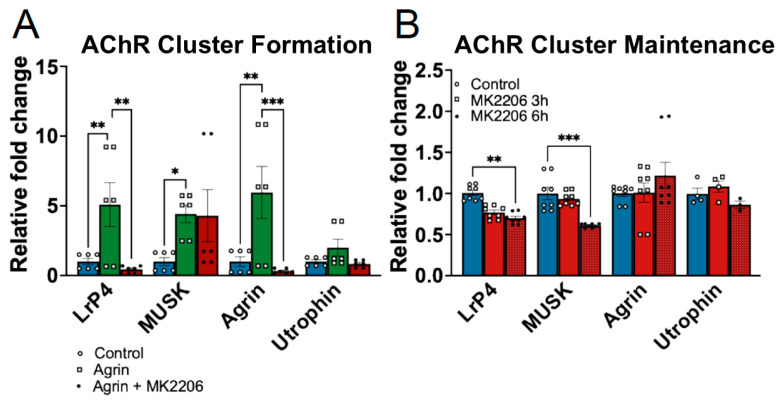
**AKT regulates NMJ-associated gene transcription during both formation and maintenance of AChR clusters.** (**A**) qPCR analysis of NMJ-associated genes (LRP4, MuSK, Agrin, and Utrophin) during the formation phase. (**B**) qPCR analysis of NMJ-associated genes during the formation and maintenance phase. mRNA expression was normalized to the housekeeping gene (TBP-1) and expressed as fold change relative to control. Data are presented as mean ± SEM. * *p* < 0.05, ** *p* < 0.01, *** *p* < 0.001 vs. unstimulated control or Agrin treatment.

During the formation phase, Agrin stimulation alone induced a significant increase in the transcript levels of *LRP4*, *MUSK*, and *AGRN*, which encode for LRP4, MUSK, and Agrin, respectively. Pretreatment with MK2206 for 30 min significantly reduced Agrin-induced *LRP4* and *AGRN* transcription, while MUSK expression remained unchanged. No significant changes were observed in *UTRN* (encoding for Utrophin) transcript levels under either condition (Figure 3A). These findings suggest that AKT selectively modulates a subset of NMJ-associated genes during the initial phase of AChR cluster formation.

During the maintenance phase, short-term AKT inhibition (3 h) did not significantly affect the expression of NMJ-associated genes. In contrast, extending MK2206 treatment to 6 h during the final phase of Agrin stimulation resulted in a significant reduction in *LRP4* and *MUSK* transcript levels, while *AGRN* and *UTRN* expression remained unchanged (Figure 3B).

The temporal pattern of transcriptional regulation suggests that early reductions in AChR cluster intensity following AKT inhibition are unlikely to be driven by immediate transcriptional suppression of NMJ genes. Instead, prolonged AKT inhibition appears to impact both receptor stability and transcriptional maintenance of key postsynaptic components.

Together, these findings indicate that AKT signaling regulates distinct sets of NMJ-associated genes during both the formation and maintenance of AChR clusters, with LRP4 emerging as a consistent AKT-dependent target in both phases.

### 3.4. AKT Maintains Agrin-Mediated AChR Intensity via the Proteasome Degradation Pathway

Because pharmacological inhibition of AKT markedly reduced Agrin-induced AChR-associated fluorescence intensity, we hypothesized that AKT may regulate AChR stability by limiting proteasome-dependent degradation. To test this possibility, C2C12 myotubes were treated with MG132, a well-established proteasome inhibitor, in combination with MK2206.

**Figure 4:**
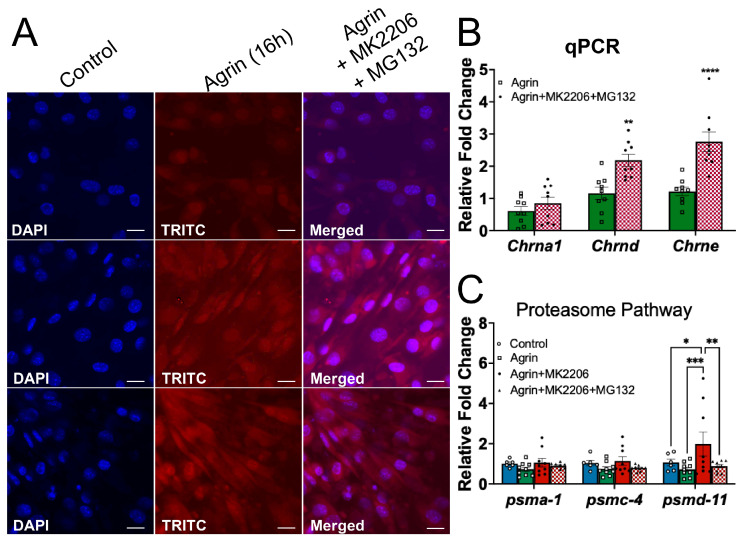
**AKT maintains Agrin-mediated AChR clusters via the proteasome degradation pathway.** (**A**) Representative confocal images of AChR clusters labeled with α-bungarotoxin. MK2206 treatment (6 h) markedly reduced AChR cluster intensity, whereas pretreatment with the proteasome inhibitor MG132 (10 μM, 30 min) restored cluster abundance. (**B**) qPCR analysis of AChR transcripts in C2C12 myotubes treated with Agrin or Agrin ± MK2206 and MG132. (**C**) qPCR analysis of proteasome-related genes (*Psma1*, *Psmc4*, and *Psmd11*) in C2C12 myotubes treated with Agrin ± MK2206 or Agrin + MK2206 and MG132. Data are presented as mean ± SEM. * *p* < 0.05 vs. unstimulated control, ** *p* < 0.01 vs. unstimulated Agrin, *** *p* < 0.001 vs. Agrin+MK2206. **** *p* < 0.0001. Images were acquired using a 40× objective. Images were acquired using a 40× objective; scale bar, 20 µm.

As expected, pretreatment with MG132 for 30 min prior to MK2206 exposure for 6 h prevented the MK2206-mediated reduction in AChR-associated fluorescence intensity, resulting in AChR intensity levels comparable to those observed in Agrin-treated cells alone (Figure 4A and Supplementary Figure S3A), indicating that proteasome inhibition rescues the loss of AChR clusters induced by AKT blockade. Importantly, despite restoring AChR fluorescence intensity, MG132 pretreatment did not rescue the MK2206-mediated reduction in *Chrna1*, *Chrnd*, or *Chrne* transcript levels (Figure 4B and Supplementary Figure S3A). This dissociation between receptor intensity and transcript abundance suggests that the protective effect of MG132 occurs at the post-translational level rather than through restoration of AChR gene expression.

To further determine whether AKT inhibition alters the proteasome pathway, we examined the expression of representative proteasome-related genes (*psma1*, *psmc4*, and *psmd1*). No significant changes were observed in the transcription of these genes following MK2206 treatment. Furthermore, MG132 pretreatment did not produce consistent transcriptional changes in these proteasome subunits (Figure 4C and Supplementary Figure S3A), indicating that the rescue effect is unlikely to be mediated through transcriptional regulation of proteasome components.

Collectively, these findings support a model in which AKT activity maintains Agrin-induced AChR cluster intensity by limiting proteasome-dependent degradation of postsynaptic receptors. The ability of MG132 to restore AChR fluorescence without correcting transcript levels further indicates that AKT primarily regulates receptor stability through post-translational mechanisms.

## 4. Discussion

The stability of AChR clusters at the NMJ is essential for efficient synaptic transmission and muscle function, yet the signaling pathways that govern their maintenance remain incompletely defined. In this study, we identify AKT as a central regulator of both the formation and maintenance of Agrin-induced AChR accumulation in C2C12 myotubes, thereby providing new insight into the molecular control of postsynaptic NMJ homeostasis.

We demonstrate that the pharmacological inhibition of AKT activity with MK2206 significantly attenuates Agrin-induced AChR surface intensity in non-permeabilized C2C12 myotubes, as measured by immunocytochemistry, and was associated with the decreased expression of AChR subunit transcripts (Figure 1 and Figure 3). These findings are consistent with the previous studies, which demonstrate that the ErbB4/NRG-1-AKT/mTOR axis regulates AChR expression [25], establishing the role of AKT in AChR stability and postsynaptic regulation.

Our time-course study reveals that short-term AKT inhibition for 3 h resulted in reduced surface intensity, with effects becoming more pronounced after 6 h (Figure 2). Transcriptionally, AKT inhibition during the maintenance phase resulted in reduced transcription of *LRP4* and *MUSK* (Figure 2 and Figure 3), suggesting that AKT signaling is required to maintain the Agrin–LRP4–MuSK signaling axis after initial AChR accumulation. These findings are consistent with prior work showing that AKT/mTOR signaling is required for NMJ gene regulation during muscle denervation [19], supporting the role of AKT in preserving postsynaptic receptor integrity following formation. Because we did not directly assess LRP4 or Agrin protein abundance, we cannot determine whether reduced transcript levels translate into decreased protein expression or impaired receptor–ligand interactions at the postsynaptic membrane. Therefore, while the transcriptional suppression of these genes likely contributes to the impaired AChR clustering observed during the formation phase, a direct causal relationship between reduced LRP4/AGRN protein levels and diminished clustering remains to be established. Future studies examining protein abundance, receptor localization, and MuSK activation will be required to clarify this relationship.

Mechanistically, we demonstrate that the inhibition of the proteasome pathway with MG132 rescues AChR surface intensity in the context of AKT inhibition, despite the persistent suppression of AChR subunit transcripts (Figure 4). This dissociation between transcriptional downregulation and restored receptor intensity suggests that AKT maintains AChR stability, at least in part, through post-translational mechanisms involving proteasome-dependent degradation. Interestingly, no significant changes were observed in any of the genes associated with the proteasome component pathway (*psma1*, *psmc4* and *psmd11*) following AKT inhibition (Figure 4), indicating that enhanced receptor loss is unlikely to result from transcriptional upregulation of core proteasome subunits. Instead, AKT may regulate proteasome activity through post-transcriptional or functional mechanisms rather than altering expression of proteasome machinery. We acknowledge, however, that MG132 has known off-target effects beyond proteasome inhibition. While the restoration of AChR surface intensity by MG132 is consistent with a proteasome-dependent mechanism, we cannot completely exclude alternative effects of MG132 on other proteolytic or signaling pathways. Future studies using complementary strategies—including alternative proteasome inhibitors, genetic suppression of proteasome components, direct measurement of proteasome activity, and assessment of AChR ubiquitination—will be necessary to definitively establish the specificity of this pathway.

While our findings highlight a proteasome-dependent mechanism by which AKT maintains AChR stability, we cannot rule out contributions from lysosomal or autophagic pathways. Previous studies have indicated that autophagy and lysosomal degradation can influence AChR turnover and postsynaptic receptor maintenance at the NMJ [26,27,28]. Determining whether AKT also modulates these pathways, and how proteasomal and lysosomal mechanisms may intersect to regulate postsynaptic receptor homeostasis, will be an important direction for future investigation.

In addition to restraining the proteasome-mediated degradation of AChR clusters, AKT signaling also intersects with known mechanisms of AChR delivery at the postsynaptic membrane. Previous work has shown that Agrin activation of PI3K leads to downstream phosphorylation and inactivation of GSK3β, which enhances the binding of the microtubule plus-end tracking protein CLASP2 to microtubules and promotes focal delivery of AChRs to synaptic sites via microtubule capture [29]. Agrin-stimulated PI3K/AKT signaling, therefore, supports AChR cluster growth and stability by facilitating both receptor insertion through microtubule-dependent trafficking and receptor retention by limiting proteasomal removal. This suggests that AKT may coordinate multiple, parallel processes that tune surface receptor density—balancing delivery with controlled degradation—to maintain NMJ integrity. Future work examining how AKT modulates GSK3β-/CLASP2-mediated delivery alongside proteasome activity will further clarify how these pathways collectively regulate postsynaptic receptor homeostasis.

Importantly, Agrin stimulation alone did not elicit detectable changes in AKT phosphorylation or downstream signaling, indicating that AKT does not function as a direct downstream effector of Agrin signaling (Figure 1). These findings suggest that AKT signaling is not directly engaged by Agrin but instead acts upstream or in parallel to the Agrin–LRP4–MuSK axis, potentially by maintaining transcriptional competence, restraining proteasome activity, or stabilizing postsynaptic protein complexes. This distinction refines the current model of NMJ regulation: rather than acting as a classical downstream effector of Agrin, AKT serves as a permissive intracellular regulator that supports postsynaptic maintenance independently of direct Agrin-induced activation.

Given that AKT signaling is dysregulated in conditions such as sarcopenia, Duchenne muscular dystrophy, and amyotrophic lateral sclerosis (ALS) [19], impaired AKT activity may contribute to postsynaptic instability and NMJ degeneration in these pathologies. Future studies will focus on defining the hierarchical relationship between AKT and Agrin–LRP4–MuSK signaling, to determine whether muscle-specific modulation of AKT signaling influences NMJ morphology and function, and whether targeting the AKT–proteasome axis can be exploited therapeutically to preserve NMJ integrity during aging or neuromuscular disease.

In summary, our findings identify AKT as a critical regulator of AChR stability through dual mechanisms: sustaining the transcription of NMJ-associated genes and restraining the proteasome-dependent degradation of postsynaptic receptors. These results highlight AKT as an important integrator of metabolic signaling and synaptic maintenance and provide new mechanistic insight into the intracellular pathways governing NMJ homeostasis.

## Data Availability

The data presented in this study are available within the article and its Supplementary Materials.

## Supplementary Materials

The following supporting information can be downloaded at: https://www.mdpi.com/article/10.3390/medicina62030456/s1, Figure S1: Quantification of Western blot and surface AChR fluorescence in differentiated C2C12 myotubes following Agrin stimulation with or without AKT inhibition. C2C12 myotubes were pre-treated with the AKT inhibitor MK2206 (10 μM, 30 min) and subsequently stimulated with neural Agrin for 16 h. Surface AChRs were labeled under non-permeabilized conditions using TRITC–α-bungarotoxin. ((A) Quantification of the Western blot shown in Figure 1B. The graph represents the ratio of phosphorylated S6 (pSer240/244S6) normalized to HSP90. (B) Surface AChR fluorescence intensity was quantified from multiple random fields per condition using ImageJ. Data are presented as mean ± SEM from 4–5 independent experiments. Figure S2: Time-dependent effect of AKT inhibition on maintenance of Agrin-induced AChR surface intensity. Differentiated C2C12 myotubes were treated with neural Agrin (16 h) to induce AChR clustering. MK2206 (10 μM) was applied during the final 3 h or 6 h of Agrin stimulation. Surface AChRs were labeled with Alexa-647–conjugated α-bungarotoxin under non-permeabilized conditions. (A) Mean fluorescence intensity was quantified using ImageJ from multiple random fields per condition. Data are expressed as mean ± SEM from 4–5 independent experiments. Figure S3: Quantification of surface AChR fluorescence intensity following AKT inhibition with or without proteasome blockade. Differentiated C2C12 myotubes were treated with neural Agrin (16 h). MK2206 (10 μM) was applied during the final 6 h of Agrin stimulation, with or without 30 min pretreatment with the proteasome inhibitor MG132. Surface AChRs were labeled under non-permeabilized conditions using fluorescent α-bungarotoxin. (A) Mean fluorescence intensity per myotube was quantified using ImageJ from multiple random fields per condition. Data are presented as mean ± SEM from 4–5 independent experiments and expressed as mean fluorescence intensity.
