# Supplementary material for: AKT Signaling Regulates Agrin-Mediated Acetylcholine Receptor Surface Density
Source: Medicina (Kaunas). 2026 Feb 27;62(3):456. doi: 10.3390/medicina62030456 (PMC13028288; doi:10.3390/medicina62030456)
Supplement: Supplementary file 1 [file medicina-62-00456-s001.zip › medicina-4117472-supplementary.pdf]

## Supplementary Figure Legends

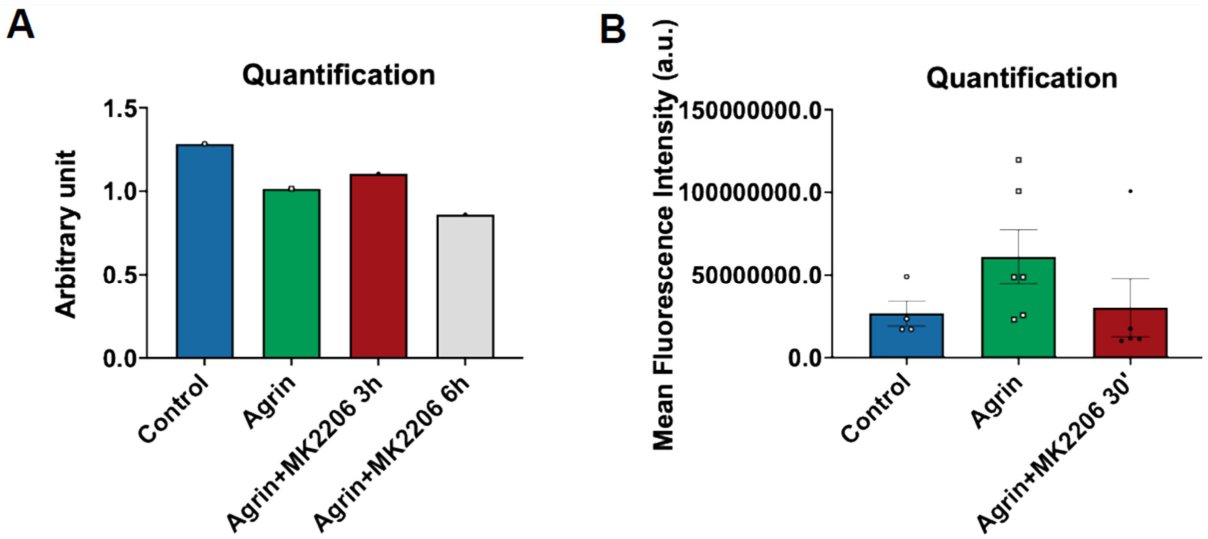


**Supplementary Figure S1.** Quantification of Western blot and surface AChR fluorescence in differentiated C2C12 myotubes following Agrin stimulation with or without AKT inhibition. C2C12 myotubes were pre-treated with the AKT inhibitor MK2206 (10 μM, 30 min) and subsequently stimulated with neural Agrin for 16 h. Surface AChRs were labeled under non-permeabilized conditions using TRITC–$\alpha$-bungarotoxin. ((A) Quantification of the Western blot shown in Figure 1B. The graph represents the ratio of phosphorylated S6 (pSer240/244S6) normalized to HSP90. (B) Surface AChR fluorescence intensity was quantified from multiple random fields per condition using ImageJ. Data are presented as mean ± SEM from 4–5 independent experiments.

A

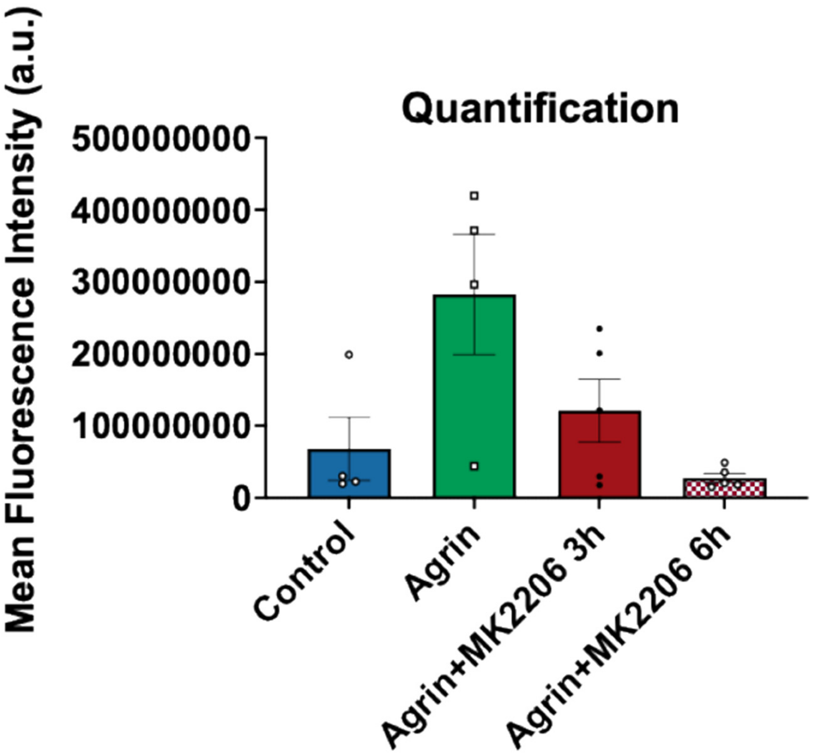


**Supplementary Figure S2.** Time-dependent effect of AKT inhibition on maintenance of Agrin-induced AChR surface intensity. Differentiated C2C12 myotubes were treated with neural Agrin (16 h) to induce AChR clustering. MK2206 (10 μM) was applied during the final 3 h or 6 h of Agrin stimulation. Surface AChRs were labeled with Alexa-647–conjugated $\alpha$-bungarotoxin under non-permeabilized conditions. (A) Mean fluorescence intensity was quantified using ImageJ from multiple random fields per condition. Data are expressed as mean ± SEM from 4–5 independent experiments.

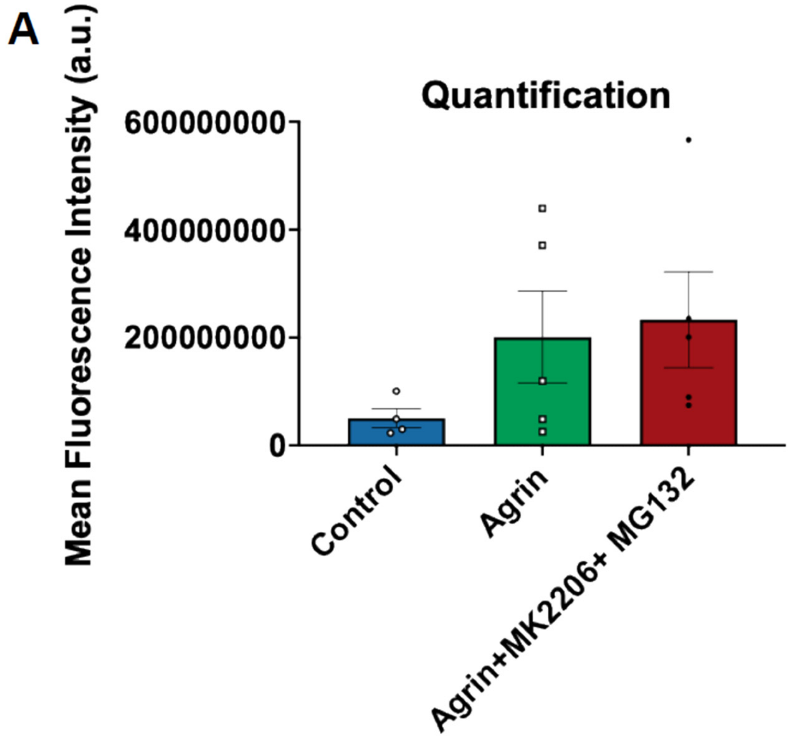


**Supplementary Figure S3.** Quantification of surface AChR fluorescence intensity following AKT inhibition with or without proteasome blockade. Differentiated C2C12 myotubes were treated with neural Agrin (16 h). MK2206 (10 μM) was applied during the final 6 h of Agrin stimulation, with or without 30 min pretreatment with the proteasome inhibitor MG132. Surface AChRs were labeled under non-permeabilized conditions using fluorescent $\alpha$-bungarotoxin. (A) Mean fluorescence intensity per myotube was quantified using ImageJ from multiple random fields per condition. Data are presented as mean ± SEM from 4–5 independent experiments and expressed as mean fluroscence intensity.
